# Colonization with *Staphylococcus aureus* and *Klebsiella pneumoniae* causes infections in a Vietnamese intensive care unit

**DOI:** 10.1099/mgen.0.000514

**Published:** 2021-01-27

**Authors:** Duong Bich Thuy, James Campbell, Cao Thu Thuy, Nguyen Van Minh Hoang, Phat Voong Vinh, To Nguyen Thi Nguyen, Chau Nguyen Ngoc Minh, Duy Thanh Pham, Maia A. Rabaa, Nguyen Phu Huong Lan, Nguyen Van Hao, Guy E. Thwaites, C. Louise Thwaites, Stephen Baker, Nguyen Van Vinh Chau, Hao Chung The

**Affiliations:** ^1^​ The Hospital for Tropical Diseases, Ho Chi Minh City, Vietnam; ^2^​ Oxford University Clinical Research Unit, Ho Chi Minh City, Vietnam; ^3^​ Centre for Tropical Medicine and Global Health, Nuffield Department of Clinical Medicine, Oxford University, Oxford, UK; ^4^​ Cambridge Institute of Therapeutic Immunology & Infectious Disease (CITIID), University of Cambridge, Cambridge, UK

**Keywords:** colonization, hospital-acquired infections, hypervirulent *Klebsiella pneumoniae*, intra-patient diversity, *Staphylococcus aureus*

## Abstract

Pre-existing colonization with *Staphylococcus aureus* or *Klebsiella pneumoniae* has been found to increase the risk of infection in intensive care patients. We previously conducted a longitudinal study to characterize colonization of these two organisms in patients admitted to intensive care in a hospital in southern Vietnam. Here, using genomic and phylogenetic analyses, we aimed to assess the contribution these colonizing organisms made to infections. We found that in the majority of patients infected with *S. aureus* or *K. pneumoniae *, the sequence type of the disease-causing (infecting) isolate was identical to that of corresponding colonizing organisms in the respective patient. Further in-depth analysis revealed that in patients infected by *S. aureus* ST188 and by *K. pneumoniae* ST17, ST23, ST25 and ST86, the infecting isolate was closely related to and exhibited limited genetic variation relative to pre-infection colonizing isolates. Multidrug-resistant *S. aureus* ST188 was identified as the predominant agent of colonization and infection. Colonization and infection by *K. pneumoniae* were characterized by organisms with limited antimicrobial resistance profiles but extensive repertoires of virulence genes. Our findings augment the understanding of the link between bacterial colonization and infection in a low-resource setting, and could facilitate the development of novel evidence-based approaches to prevent and treat infections in high-risk patients in intensive care.

## Data Summary

Raw sequencing data used in this study are available under the NCBI Bioproject accession PRJNA646358. The metadata for sequenced *S. aureus* and *K. pneumoniae *, together with their phylogenies, have been uploaded in Figshare (https://figshare.com/projects/Colonization_with_Staphylococcus_aureus_and_Klebsiella_pneumoniae_cause_infections_in_a_Vietnamese_intensive_care_unit/88109).

**Impact Statement**

Bacteria, including *Staphylococcus aureus* and *Klebsiella pneumoniae *, frequently colonize and thrive in the respiratory and gastrointestinal tracts of humans. Colonization with such bacteria could predispose vulnerable individuals to diseases, particularly patients admitted to intensive care units (ICUs). High-resolution genomic approaches have been utilized to unravel the genetic relationship between colonizing and infecting bacteria in ICU patients, mostly in high-income settings. However, data concerning this colonization–infection causality is limited in low-resource ICU settings, such as in Vietnam. Here, we combined detailed clinical investigation and genomic analyses to tackle this question. We found that in the majority of patients, the bacteria isolated from the infection site were closely related to those colonizing the respective patient prior to disease onset, confirming that the illness was caused by pre-existing colonizing *S. aureus* or *K. pneumoniae *. We also identified the predominance of a multidrug-resistant *S. aureus* clone (ST188), and several hypervirulent *K. pneumoniae* clones among the documented infections. Our finding suggests that the patients’ own microbiome could serve as a reservoir for pathogens, and that interventions to monitor and/or alter bacterial colonization should be considered to minimize the risk of infection in these patients.

## Introduction

The intensive care unit (ICU), although providing critical care and life support for critically ill patients, is the major source of hospital-acquired infections (HAIs), with the risk of infection 3–10 times higher than that in general wards [1, 2]. Patients admitted to the ICU often suffer from co-morbidities, which may increase the duration of hospitalization, cost of treatment and mortality. ICUs are becoming increasingly integrated into healthcare systems of low- and middle-income countries (LMICs), but limited data on ICU-associated infections are available from such settings. According to the World Health Organization (WHO) in 2010, only 23 LMICs (15.6 %) reported a functional national surveillance system for HAIs [3]. Therefore, understanding the burden and nature of ICU-associated infections remains a priority in LMICs in order to prevent infections, improve treatment and reduce mortality rates.

The majority of reported HAIs are of bacterial aetiology, with pathogen distributions varying temporally and geographically. However, *Staphylococcus aureus* and *Klebsiella pneumoniae* are consistently associated with HAIs, and antimicrobial resistance (AMR) severely limits treatment options in infections caused by these pathogens [4]. The Gram-positive *S. aureus* frequently colonizes the nares (nostrils), pharynx and skin. Carriage of *S. aureus* is generally not harmful, but it poses a significant risk of infection in vulnerable patients, including skin and soft tissue infection (SSTI), arthritis, endocarditis, pneumonia and bloodstream infection (BSI) [5, 6]. Most *S. aureus* isolates are susceptible to methicillin (MSSA), a key empirical antimicrobial. However, infections by *S. aureus* have been complicated by the emergence of methicillin-resistant *S. aureus* (MRSA), associated with both serious HAIs and community-acquired infections (CAIs) [7]. The Gram-negative Enterobacteriaceae *K. pneumoniae* can also asymptomatically colonize the skin, nares, mouth, pharynx and gastrointestinal tract of humans. Serious *K. pneumoniae* infections include SSTI, cholecystitis, liver abscess, meningitis, urinary tract infection (UTI), pneumonia and BSI [8]. Recently, multidrug-resistant (MDR) *K. pneumoniae *, including those resistant to the last-resort antimicrobial classes carbapenems and polymyxins (colistin), have emerged as important nosocomial pathogens with associated high morbidity and mortality [9–12].

We previously conducted a study in the adult ICU at the Hospital for Tropical Diseases (HTD) (a tertiary infectious diseases referral hospital) in Ho Chi Minh City (HCMC), Vietnam, to characterize bacterial colonization and infections in adult inpatients [13, 14]. We found that 13.1 % (110/838) of patients carried *S. aureus* in their nares upon ICU admission, of whom 65.5 % were MRSA carriers [13]. All patients with *S. aureus* HAIs (*n*=10) were found to have been colonized with the pathogen prior to infection [14]. Similarly, HAIs caused by *K. pneumoniae* were preceded by positive *K. pneumoniae* cultures from the same patient (17/18 cases) [14].

Our findings suggested that prior colonization by these two bacteria was associated with subsequent infection in ICU patients. However, conventional microbiological approaches were unable to resolve the relationships between colonizing and infecting bacteria. Molecular methods offer the opportunity to establish the genetic relatedness between colonizing and infecting *S. aureus* and *K. pneumoniae* in individual patients [6, 15–20]. Here, we aimed to test the hypothesis that colonization with *S. aureus* and *K. pneumoniae* leads to an infection caused by these organisms in ICU patients (including both CAIs and HAIs), as well as to explore the pathogens’ genetic determinants of AMR and virulence. To address these aims, we measured the genetic relatedness between colonizing and infecting *S. aureus* and *K. pneumoniae* isolates using whole-genome sequencing (WGS) and phylogenetic analysis.

## Methods

### Study design and sample collection

This prospective cohort study enrolled patients (≥15 years old) hospitalized in the adult ICU at the HTD from 10 November 2014 to 14 January 2016 [13, 14]. The study received ethical approval from the Ethics Committee of the HTD, Vietnam, and the Oxford University Tropical Research Ethics Committee (OxTREC), UK. Written informed consent was obtained from all recruited patients or their legal representatives. All eligible patients had a nasal swab, a rectal swab and an endotracheal aspirate (ETA, in case of intubation or tracheostomy) taken within 48 h of ICU admission, and this sampling scheme was repeated twice a week to screen for *S. aureus* and/or *K. pneumoniae* colonization until the patients were discharged from the ICU. Patients were then monitored to record the development of infections, and the ascertainment of *S. aureus* and *K. pneumoniae* infections was performed by ICU doctors according to the Centers for Disease Control and Prevention (CDC) guidelines [21]. These infections were further categorized into three groups, including CAIs, HAIs and healthcare-associated infections (HCAIs), based on the time of disease onset after hospital admission and the history of hospitalization [22, 23].

Microbiological methods utilized in this study have been described previously [13, 14]. In brief, nasal and endotracheal specimens were cultured on blood agar and MacConkey agar, while rectal swabs were cultured on MacConkey agar to isolate *S. aureus* and *Klebsiella* species. Recovered colonies were subjected to conventional biochemical identification tests, and isolate identities were confirmed on a MALDI-TOF mass spectrometer (Bruker). Due to resource constraints, a maximum of two confirmed *S. aureus* or *K. pneumoniae* colonies (in the case of differing morphology) were stored from any positive culture plate. All consecutive, non-duplicate *S. aureus* and *K. pneumoniae* isolates, including colonizing and infecting isolates, obtained from patients with confirmed diagnoses of *S. aureus* and *K. pneumoniae* infections were included in this study. The infecting isolates were recovered from clinical specimens during an infection episode; clinical specimen types include pus sample (in the case of SSTI), sputum or ETA (pneumonia), blood sample (BSI), urine sample (UTI) and peritoneal fluid sample (SBP). The colonizing isolates were obtained via culturing from swabs (nasal swab, rectal swab and/or ETA) taken prior to the timepoint of *S. aureus* or *K. pneumoniae* infections being diagnosed.

### Antimicrobial susceptibility testing

Antimicrobial susceptibility testing was conducted by the Kirby/Bauer disc diffusion method and interpreted using the Clinical and Laboratory Standards Institute (CLSI) guidelines 2015 [24]. For *S. aureus *, we performed testing for the following antimicrobials: penicillin, oxacillin, vancomycin, erythromycin, rifampicin, clindamycin, ciprofloxacin and trimethoprim-sulfamethoxazole (co-trimoxazole). Additionally, bacteraemia-associated *S. aureus* were subjected to testing for susceptibility to linezolid and teicoplanin. For *Klebsiella* species, the following antimicrobials were tested: ceftriaxone, cefepime, meropenem, amikacin, ciprofloxacin, piperacillin/tazobactam, ticarcillin/clavulanate and colistin. For both pathogens, MDR was defined as non-susceptibility to ≥1 agent in ≥3 antimicrobial classes, while extreme-drug resistance (XDR) was defined as non-susceptibility to ≥1 agent in all but two or fewer antimicrobial classes.

### WGS and phylogenetic reconstruction

DNA was extracted from pure recovered bacterial isolates (67 *S *. *aureus* and 141 *K *. *pneumoniae *) using the Wizard Genomic DNA Purification Kit (Promega), following the manufacturer’s instructions. WGS was performed on the in-house Illumina MiSeq bench-top sequencer (for *S. aureus *) or via a commercial partner (Macrogen; for *K. pneumoniae *), using the Nextera DNA Library Prep kit (Illumina). Data analysis was performed on the Cloud Infrastructure for Microbial Genomics (CLIMB) [25] and on the OUCRU’s computational cluster. Sequencing quality was checked by FASTQC, and multi-locus sequence typing (MLST) was conducted using ARIBA [26]. For *S. aureus *, the MLST results indicated that ST188 was the predominant sequence type (ST) in this study (39/67), so reference-based mapping was further conducted for 39 samples of ST188, against the reference MSSA476 (accession number: NC_002953) using bwa-mem with default setting [27]. This reference, which belongs to ST1, was selected because it was the most closely related complete reference to ST188 at the time of analysis. Nine *S. aureus* isolates were classified as *Staphylococcus argenteus* by MLST (ST2250 and ST1223), and these were mapped against the complete reference *S. argenteus* ASM23692 (accession number: NC_016941) using bwa-mem (default setting). For *K. pneumoniae *, only ST17, ST23, ST86 and ST25 were included for further mapping analyses because they were isolated from ≥3 ICU patients or intensively sampled within one patient (ST25: 18 isolates). For each of these STs, short reads were mapped to the appropriate reference (XH209, accession number NZ_CP009461 for ST17; NTUH-K2044, accession number NC_012731 for ST23; CG43, accession number NC_022566 for ST86; SMU18037509, accession number NZ_CP045661 for ST25) using bwa-mem (default setting) [27].

SNPs against the reference were detected and filtered using SAMtools (v1.3) and bcftools (v1.2), respectively [28]. Duplicate reads were removed by PICARD, and the package GATK was employed for indel realignment, as previously recommended [29]. SNPs were called using the ‘consensus’ option, and low-quality SNPs were removed if they met any of the following criteria: consensus quality <50, mapping quality <30, ratio of SNPs to reads at a position <90 % and read depth <4. This helps to create a set of high-quality SNPs suitable for investigating patient-to-patient transmission. Mapping coverage at each position in the reference genome was summarized using bedtools (v2.24.0). A pseudo-sequence (with the same length of the mapping reference) was created to incorporate the identified SNPs, regions of low mapping coverage and invariant sites, using the vcf2fa python script (https://github.com/brevans/vcf2fa; --min_cov=4). Pseudo-sequences of the same ST were concatenated to create alignments suitable for phylogenetic reconstruction. Exclusion of genomic regions pertaining to recombination or horizontal gene transfer (such as prophages, genomic islands) was performed using Gubbins, with default setting and ten iterations to ensure convergence [30]. Removal of invariant sites further generated alignments of 1005 bp for *S. aureus* ST188, 244 bp for *S. argenteus* ST2250, 781 bp for *K. pneumoniae* ST17, 468 bp for *K. pneumoniae* ST23, 697 bp for *K. pneumoniae* ST86 and 88 bp for *K. pneumoniae* ST25. These alignments served as inputs for phylogenetic reconstruction of *S. aureus* and *K. pneumoniae *, and each maximum-likelihood phylogeny was inferred using RAxML v8.1.3 with the GTRGAMMA model with 1000 rapid bootstraps [31]. To assess the performance of Gubbins, ClonalFrameML (branch extension model; kappa=3.423 as inferred by PhyML; emsim=100) was used additionally to detect and remove recombination regions on the pseudo-alignment of *S. aureus* ST188 [32]. This produced an alignment of 1004 bp, and the calculated pairwise SNP difference was not significantly different from that generated using Gubbins-processed alignment. Comparison in the recombination-free SNP alignments generated by the two methods showed that they shared 838/1005 common patterns (alignment columns), which indicated the relatively high concordance of the two approaches.

### Determination of accessory genome

For all *S. aureus* and *K. pneumoniae* genomes, we separately constructed *de novo* assemblies for each read set using SPAdes v3.12.0 with default parameters [33]. Prior to assembly, each read set was processed using Trimmomatic to retain high-quality read pairs of at least 50 bp (slidingwindow: 10 : 20, paired-end option) [34]. Annotation for each assembly was determined using Prokka [35], and Roary (-i 95) was used to construct the pangenome separately for genomes of each ST to explore the genetic variation within each ST and patient [36]. To identify contigs associated with accessory genomes, the assembly was first ordered with the appropriate chromosome reference (that has been used for mapping analysis) using ABACAS (default parameters) [37]. Contigs identified to belong to plasmids were queried against the public nucleotide database (NCBI) using blastn or the bacterial plasmid resource PLSDB (https://ccb-microbe.cs.uni-saarland.de/plsdb/) [38]. Artemis and Artemis Comparison Tool (ACT) were used to visualize the presence of specific genetic elements in the isolates [39]. Additionally, all *S. aureus* assemblies were input into FastANI to compute whole-genome average nucleotide identity (ANI) against the *S. aureus* (MSSA476) and *S. argenteus* (ASM23692) references [40]. An accurate species assignment was made to each assembly if the ANI score against the respective reference was above 97.

AMR determinants were identified in all sequenced *S. aureus* and *K. pneumoniae* isolates by running ARIBA on the curated ResFinder database (--nucmer_min_id=95, --nucmer_min_len=80) [26, 41]. Additionally, virulence determinants were identified by running ARIBA on the vfdb_core database (with aforementioned parameters) for all *S. aureus* and *K. pneumoniae* sequencing reads [42]. Typing for Staphylococcal Cassette Chromosome *mec* (SCC*mec*) was conducted using SCC*mec*Finder (https://cge.cbs.dtu.dk/services/SCCmecFinder/) [43]. Furthermore, all 25 infecting *K. pneumoniae* isolates were screened for the presence of virulence factors (siderophores and *rmpA*), capsular K antigen, integrative conjugative elements (ICEs) and acquired resistance genes using Kleborate (default setting), with *de novo* assemblies serving as the input [44, 45]. All statistical analyses were conducted using R software (version 3.4.0).

## Results

### Patient characteristics

Between November 2014 and January 2016, 838 patients were enrolled and followed in the adult ICU of the HTD in HCMC [14]; 19 and 28 patients were diagnosed with an *S. aureus* or a *K. pneumoniae* infection, respectively. The majority of patients were <70 years of age, and had no or mild comorbidities [Charlson Comorbidity index (CCI) score <3] (Table 1). The most common reasons for ICU admission were tetanus, followed by sepsis or septic shock. Mortality associated with *K. pneumoniae* infection was higher than that of *S. aureus* (28.6 % vs 15.8 %); a comparable trend was observed with the length of ICU stay for survivors (25.5 vs 18 days). The prospective study design permitted additional sampling (at several body sites) in patients from their ICU admission until the onset of infection. The infecting and colonizing isolates recovered from the aforementioned *S. aureus* and *K. pneumoniae* infections were included for downstream analysis in order to explore potential links between colonization and infection.

**Table 1.** Characteristics of ICU patients with *S. aureus* and *K. pneumoniae* infections

Characteristic	Statistics∗	
	* S. aureus* infections (*n*=19)	* K. pneumoniae* infections (*n*=28)
Age (years)	46 (32.5–62)	59 (45–66)
Male	13 (68.4)	20 (71.4)
CCI score	0 (0–1)	0 (0–1.3)
No comorbidity (0)	13 (68.4)	17 (60.7)
Mild (1–2)	4 (21.1)	5 (17.9)
Moderate (3–4)	0	3 (10.7)
Severe (≥5)	2 (10.5)	3 (10.7)
APACHE II score	11 (4–19)	10.5 (7–19)
Mild (<5)	7 (36.8)	4 (14.2)
Moderate (5–12)	4 (21.1)	12 (42.9)
Severe (>12)	8 (42.1)	12 (42.9)
Admitting diagnosis		
Tetanus	10 (52.5)	13 (46.4)
Sepsis and septic shock	5 (26.3)	8 (28.6)
Severe pneumonia	1 (5.3)	2 (7.1)
Other diagnoses^†^	3 (15.8)	5 (17.8)
Types of infection		
Pneumonia	7 (36.8)	16 (57.2)
Bloodstream infection	6 (31.6)	6 (21.4)
Skin and soft tissue infection	6 (31.6)	0
Urinary tract infection	0	4 (14.3)
Spontaneous bacterial infection	0	2 (7.1)
Death	3 (15.8)	8 (28.6)
ICU stay (days) for expired patients	7 (4–43)	5.5 (2.8–26.5)
ICU stay (days) for survivors	18 (9.8–27.3)	25.5 (6.8–32.5)
Hospital stay (days) for survivors	28 (21.5–43.3)	36.5 (16.5–42)

*Median (interquartile) for continuous variables, and *n* (%) for categorical variables.

†Other diagnoses include: severe dengue infection, hepatic encephalopathy, status epilepticus, and urinary tract infection.

APACHE II score: Acute Physiology and Chronic Health Evaluation II score.

CCI score: Charlson Comorbidity Index score.

### Identity between colonizing and infecting *Staphylococcus aureus*

**Fig. 1.**
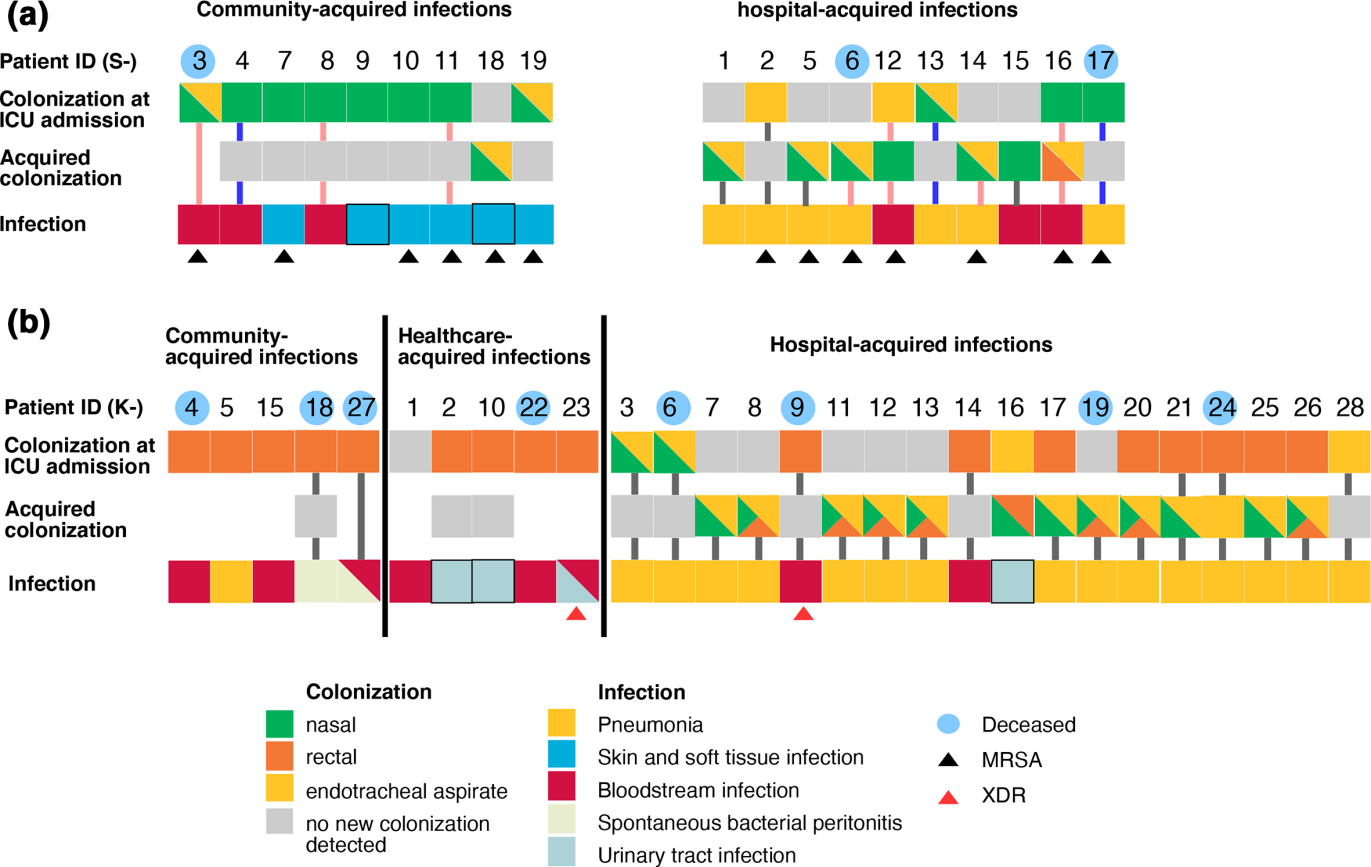
Schematic graph showing the colonization and infection of (a) 19 patients infected by *Staphylococcus aureus* and (b) 28 patients infected by *Klebsiella pneumoniae *. For colonization upon admission or acquired during ICU stay, each square box denotes a positive (coloured) or negative (grey) culture result from the patient’s respective body sites (as seen in the key). Missing boxes indicate a loss of surveillance culture during ICU stay. For infection, the colour of each box indicates the nature of disease (see key), with black-lined boxes denoting missing infecting isolates. Black and red triangles denote the methicillin resistance and extreme-drug resistance status in *S. aureus* and *K. pneumoniae* infections, respectively. A filled line connecting the boxes in each patient indicates that the infecting and colonizing isolates are of the same ST, with pink and blue lines showing that infections are caused by ST188 *S. aureus* and *S. argenteus* respectively. Patient IDs with blue shading indicate that the patient died due to the infection during the ICU stay.

Infections caused by *S. aureus* were pneumonia (7/19), BSI (6/19) or SSTI (6/19), with comparable incidences of CAI (*n*=9) and HAI (*n*=10). Screening upon ICU admission showed that 13 patients were colonized by *S. aureus* in the nasal cavity or the endotracheal tract, with three patients colonized in both niches (Fig. 1a). One patient (S3) was discharged from the ICU within 48 h of admission, so it was possible to assess the prevalence of acquired colonization for 18 patients. During ICU stay, six patients who were initially *S. aureus* negative became colonized by the bacterium (patients S1, 5, 6, 14, 15, 18). Additionally, patients S12 and S16 became colonized by MRSA in other body sites in addition to the initial site (Fig. 1a).

We recovered 67 colonizing and infecting *S. aureus* isolates from the 19 patients for WGS. MLST analysis revealed that nine isolates were of *S. argenteus* (ST2250 and ST1223), which agreed with the results from comparative genomics approach (FastANI scores against the *S. argenteus* reference >98.7)*.* One colonizing nasal isolate produced an unusually fragmented assembly (1103 contigs) and was excluded from downstream analyses. Thus, the remaining 57 *S. aureus* were composed of 14 infecting (five pneumonia, five BSIs and four SSTIs) and 43 colonizing isolates. This collection comprised eight STs, with ST188 being the most prevalent (*n*=39, infecting=9). The remaining STs (ST5, ST45, ST7, ST97, ST1232, ST15 and a novel ST) were each represented in ≤6 isolates (Table S1, available in the online version of this article). An intra-patient comparison revealed that the ST of the infecting isolate was identical to that of the colonizing isolate prior to infection in all HAIs (*n*=10) (Fig. 1a). Conversely, ST congruity was observed in 4/7 CAI cases (with retrievable infecting isolates). These data suggest that colonizing *Staphylococcus* probably caused disease in this patient cohort.

MRSA accounted for 11/14 of the confirmed *S. aureus* infections. In these cases, vancomycin was commonly administered for treatment (8/11 documented cases). Resistance to penicillin (*n*=13), erythromycin (*n*=12), clindamycin (*n*=11) and ciprofloxacin (*n*=10) were frequently noted. In contrast, non-susceptibility to rifampicin was sparse (*n*=2), while all infecting *S. aureus* remained susceptible to vancomycin. Additionally, the six *S. aureus* associated with BSI were susceptible to linezolid and teicoplanin.

### Colonizing ST188 *S. aureus* causes disease in ICU patients

**Fig. 2.**
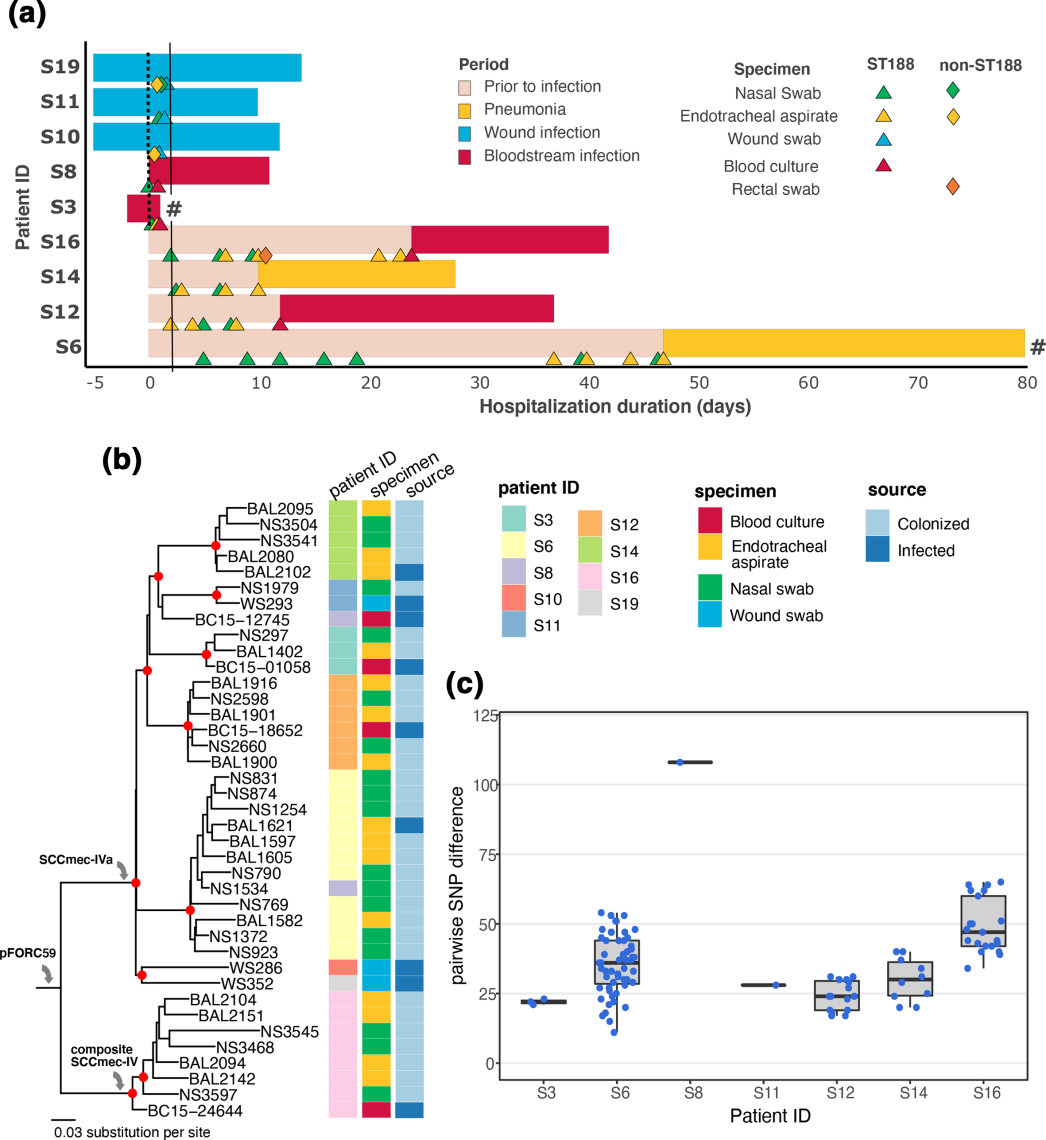
Genomic investigation of *S. aureus* ST188 causing infections in this study. (a) Schematic representation of patients’ ICU stay, with the bar colour corresponding to the infection status during or prior to the ICU stay (see key). Each triangle represents a positive culture of ST188 *S. aureus* from the patient at defined time point since ICU admission, with colour matching the type of clinical specimen (see key). Each filled diamond represents a positive non-ST188 *S. aureus* culture from the respective specimen (as coloured in the key). A ‘#’ symbol denotes that the respective patient died during the ICU stay. A solid vertical black line denotes the time point of 48 h after hospital admission. (b) Maximum-likelihood phylogeny of 39 ST188 *S. aureus* isolated in this study. The tree is mid-point rooted, and red filled circles indicate bootstrap values >80 at the internal nodes. The columns show the associated data for each taxon, including Patient ID, specimen and source of isolation (colonizing or infecting). Grey arrows denote the acquisition of accessory genetic elements into the phylogeny. (c) Dfferences (in SNPs) of ST188 *S. aureus* isolated within each patient (excluding S10 and S19), as assessed by reference-based recombination-free mapping. Each dot represents an SNP difference between two isolates, and the boxplot shows the distribution of such differences in each patient.

We next focused on patients infected by ST188 *S. aureus* to resolve the genetic relationship between colonizing and infecting isolates, by constructing a maximum-likelihood phylogeny of 39 ST188 *S. aureus *. In our cohort, ST188 caused infection in nine patients (Fig. 2a), but the colonizing isolates of two patients (S10 and S19) belonged to ST45, thus leaving only seven patients with both colonizing and infecting ST188 isolates available for inference (Fig. 2b). Five of these patients were colonized with *S. aureus* ST188 upon ICU admission, while the remainder became positive for ST188 during hospitalization and prior to developing pneumonia (Fig. 1a). Phylogenetic reconstruction delineated six highly supported clusters (bootstrap value >80), five of which exclusively contained isolates from an infected patient (S3, S11, S12, S14 and S16). The remaining cluster included all isolates from patient S6 and a nasal colonizer from patient S8. The analysis demonstrated that the infecting ST188 orgnanism was genetically closely related to the organism colonizing the same patient prior to infection on 6/7 occasions (except S8). The median pairwise SNP difference of the *S. aureus* isolates within each of these six patients ranged from 22 to 47, while its counterpart in patient S8 was 108 (Fig. 2c). This high-resolution genomic evidence implies that *S. aureus* ST188 infections were caused by the colonizing strain within the same patient in most cases, and suggests limited transmission between patients in our unit (visualized here in only one event between patients S6 and S8).

### Virulence and antimicrobial resistance in ST188 *S. aureus*

A pangenome investigation of ST188 showed little variation in the gene content of isolates from the same host (except S8; Fig. S1). Inter-patient variation stemmed mostly from prophages or plasmids. For instance, three isolates from patient S3 all carried a prophage highly similar to an *S. aureus* bovine pathogenicity island (SaPI Tokyo12381), but lacking the toxin genes (*sec* and *sel*). Genomic screening showed that ST188 carried multiple genes conferring virulence factors, with all organisms possessing haemolysins (α, δ, γ), leukocidin D (*lukD*), haem uptake system (*isdABCDEFG-srtB*) [46], serine proteases (*sspABC*, *aur*), adhesion factors (*icaABCDR*, *ebp*) and determinants involved in immune escape (*scn*, *spa, adsA, sak*). Additionally, five organisms from patient S14 acquired a prophage encoding chemotaxis-inhibiting protein (*chs*), further giving them the capacity to counter the host immune system (Fig. S1) [47]. The virulence profile of ST188 is similar to those of other infecting *S. aureus* STs (ST5, ST7, ST45 and ST97) reported herein. All ST188 isolates carried an ~35 kbp plasmid sharing high similarity and synteny with pFORC59 (accession number: NZ_CP020355), previously isolated from *S. aureus* causing BSI in South Korea. This plasmid harbours *blaZ*, *aac(6′)-Ie-aph(2′)-Ia* and *ermB*, rendering ST188 resistant to penicillin, gentamicin, erythromycin and clindamycin (Fig. S1). Furthermore, all ST188 harboured *mecA* [via SCC*mec* type IVa(2B) or composite SCC*mec* type IVhj(2B)] and double mutations (*gyrA*-S84L, *grlA*-S80F) in the quinolone resistance determining region (QRDR), which explains its resistance to methicillin and ciprofloxacin, respectively. The virulent and resistant phenotype of ST188 is reflected in two fatal infections recorded during the ICU stay, with the patients being treated with either vancomycin (S6) or ceftriaxone (S3).

### An unexpected culprit: *S. argenteus*

Genotyping revealed that two patients (S4 and S13) were infected with ST2250 organisms and one patient (S17) with ST1223 (Fig. 1a). Both STs belong to *S. argenteus *, an emerging pathogen within the *S. aureus* complex [48]. Genomic comparison showed that the colonizing and infecting isolates in each respective patient proximally clustered, indicating that the infection was probably caused by the colonizing bacterium (Fig. S2)*.* Screening for virulence determinants showed that *S. argenteus* frequently carried haemolysin-δ, *adsA* and sporadically harboured *scn* and *sak. S. argenteus* was still associated with diseases, including BSI (patient S4) and ventilator-associated pneumonia (S13 and S17). In contrast to most isolated *S. aureus *, we found that the infecting *S. argenteus* were susceptible to most tested antimicrobials, with the exception for penicillin (*n*=2) and oxacillin (*n*=1) (Fig. S2). Of note, all *S. argenteus* ST1223 from patient S17 (*n*=3) carried SCC*mec* type IVc(2B), rendering it methicillin-resistant. Thus, empirical imipenem treatment could not improve this patient’s condition, and S17 died from the infection during hospitalization.

### Identity between colonizing and infecting *K. pneumoniae*

We identified 28 *K. pneumoniae* infections, with hospital-acquired pneumonia accounting for more than half of cases (15/28) (Fig. 1b). Upon ICU admission, 75 % (21/28) of patients were colonized by *K. pneumoniae* in either the nares, endotracheal tube or rectum, with the rectum being the most common colonization site (*n*=17). Six patients were discharged from the ICU within 48 h of admission (Fig. 1b); therefore, it was only possible to assess acquired colonization in the remaining 22 patients. During the ICU stay, *K. pneumoniae* was further isolated from six patients who initially tested negative; five of these were later colonized at all three examined sites (rectal, nasal, endotracheal). *K. pneumoniae* was further identified in new body sites during their ICU stay in an additional seven patients, most commonly in the nares and endotracheal tract (Fig. 1b).

We performed WGS and analyses on all presumptive *K. pneumoniae* isolated from these patients, generating 141 sequences (27 infecting and 114 colonizing) for downstream analysis. Infecting isolates were obtained from 25 patients, with two patients (K23 and K27) having *K. pneumoniae* isolated from both blood and the infection site, while colonizing isolates were recovered from 27 patients. Preliminary MLST analyses indicated that the sequenced collection were highly diverse and comprised 28 known and five novel STs. More than one ST (two to five) was isolated from half of the patients (*n*=14). Phylogenetic reconstruction of MLST genes identified the presence of *Klebsiella quasipneumoniae* (KpII; ST1215, ST1473, ST816-1LV and two novel STs) and *Klebsiella variicola* (KpIII; ST363). Among the *K. pneumoniae* KpI, ST25 was the most common (*n*=18), followed by ST86 (*n*=17), ST420 (*n*=14), ST17 (*n*=13) and ST23 (*n*=12). Other STs were each found in ≤8 isolates. Comparable to the *Staphylococcus* infections, an intra-patient comparison showed that the majority of *Klebsiella* infections were probably caused by the patient’s colonizing isolates, as indicated by the same ST between colonizing and infecting isolates (Fig. 1b). Such concordance was observed in all HAIs (17/17 retrievable infecting isolates), but only in 2/5 CAIs and none in HCAI. Moreover, the ST of isolates recovered from blood matched that isolated from the rectal swab, while the ST of pneumonia-infecting isolates matched that of organisms colonizing the nasal or endotracheal tube (Table S2).

### Colonizing *K. pneumoniae* causes diseases in ICU patients

**Fig. 3.**
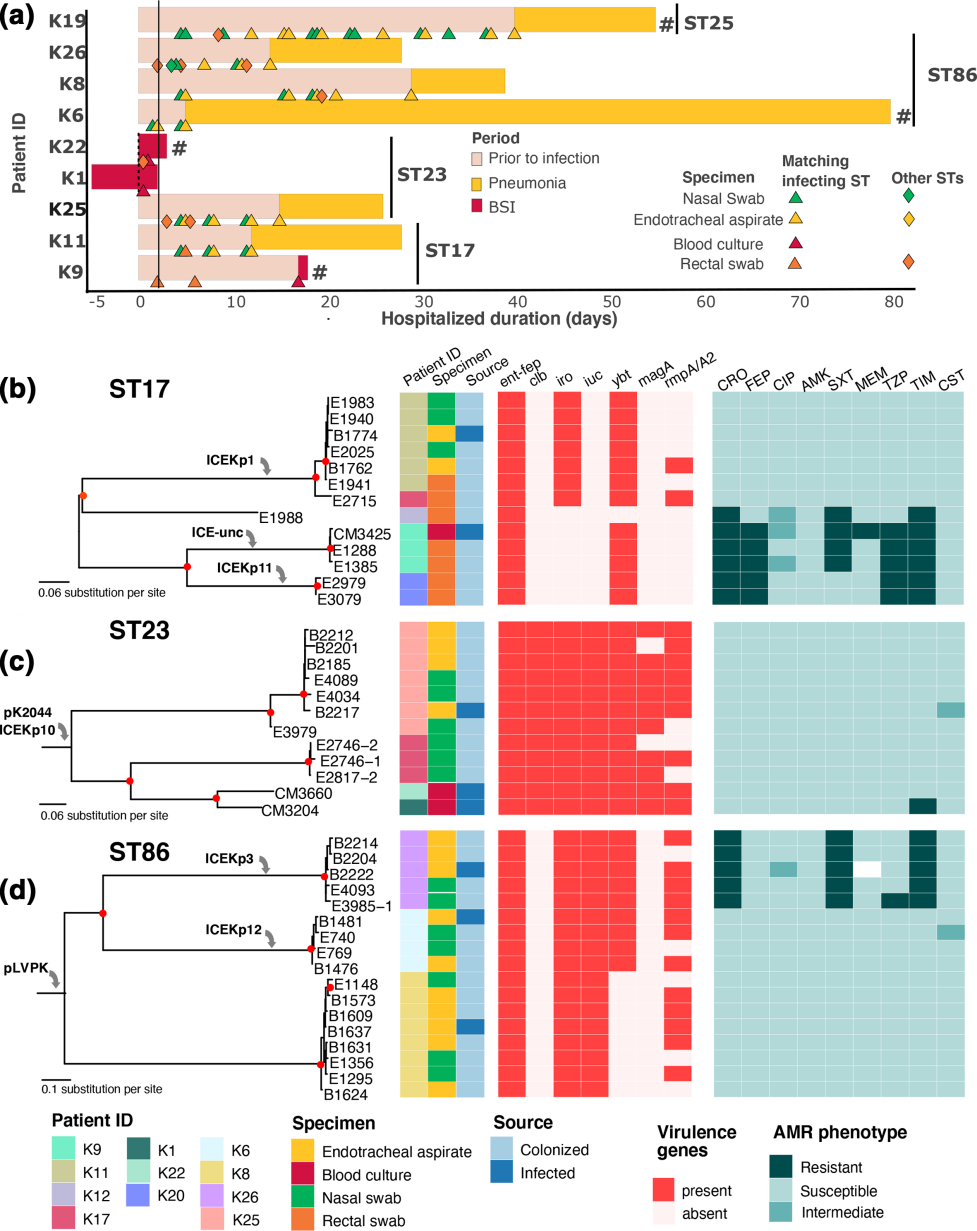
Genomic investigation of major *K. pneumoniae* STs causing infections in this study. (a) Schematic representation of patients’ ICU stay, with the bar colour corresponding to the infection status during or prior to ICU stay (see key). The vertical black line includes patients infected by the respective *K. pneumoniae* ST (ST17, ST23, ST86, ST25). Each triangle represents a positive culture of *K. pneumoniae* whose ST matches that of the infecting isolate, with colour denoting the type of clinical specimen (see key). Each filled diamond represents a positive *K. pneumoniae* culture of STs not matching that of the infecting isolate, from the specimen as coloured in the key. A ‘#’ symbol denotes that the respective patient died during the ICU stay. A solid vertical black line denotes the time point of 48 h after hospital admission. The remaining panels display the maximum-likelihood phylogeny of (b) ST17, (c) ST23 and (d) ST86 *K. pneumoniae* isolated from this study. Each tree is mid-point rooted, and red filled circles indicate bootstrap values >80 at the internal nodes. The columns show the associated information for each taxon, including Patient ID, specimen and source of isolation (colonizing or infecting); the presence of virulence genes, including *ent-fep* (enterobactin), *clb* (colibactin), *iro* (salmochelin), *iuc* (aerobactin), *ybt* (yersiniabactin), *magA* (mucoviscosity associated gene A), *rmpA/A2* (regulator of mucoid phenotype A/A2); susceptibility to antimicrobials, including CRO (ceftriaxone), FEP (cefepime), CIP (ciprofloxacin), AMK (amikacin), SXT (co-trimoxazole), MEM (meropenem), TZP (piperacillin/tazobactam), TIM (ticarcillin/clavulanate), CST (colistin). Grey arrows denote the acquisition of accessory genetic elements into the phylogeny. ICE-unc: uncharacterized ICE.

We performed a more in-depth analysis of ST17, ST23 and ST86, as these genotypes were detected in five, four and three patients (infecting and/or colonizing), respectively. ST17 was isolated from five patients, two of whom had both colonizing and infecting isolates of this ST (Fig. 3a). ST23 was detected in four patients, in which two HCA–BSI cases lacked a surveillance culture and one consisted of only nasal colonizing organisms. We retrieved both colonizing and infecting isolates of ST86 in three hospital-acquired pneumonia cases. In the HAIs, we collected several colonizing isolates (2–17 per patient) with STs matching that of the infecting isolates. Separate phylogenetic reconstructions of these genotypes revealed that each phylogenetic cluster (defined as bootstrap >80) contained isolates from each patient only (Fig. 3b–d) except for a potential transmission event of an ST17 between patients K11 and K17. The infecting isolate within each patient was genetically closely related to the colonizing isolates. We additionally conducted a genomic analysis on the ST25 organisms, which were all isolated from a single patient (infecting *n*=1; colonizing *n*=17) (Fig. 3a). The median pairwise difference among ST25 isolates was six SNPs (range: 0–38 SNPs), and phylogenetic inference failed to construct a highly resolved structure, suggesting that the colonizing *K. pneumoniae* were highly likely to have caused the infection in this patient.

### High virulence potential but low antimicrobial resistance in infecting *K. pneumoniae*

We screened all *Klebsiella* sequences for virulence genes, focusing on siderophores (iron-acquisition) and hyper-mucoviscosity (*rmpA*/*rmpA2*) [49, 50]. The majority (*n*=20) carried more than one siderophore locus (Table 2). Notably, all ST23 and one ST65 infecting isolates carried all five characterized siderophores: enterobactin (*ent-fep*), aerobactin (*iucABCD-iutA*), yersiniabactin (*ybt-irp-fuyA*), colibactin (*clbA-Q*) and salmochelin (*iroBCDE*) (Fig. 3c). Acquisition of the canonical pLVPK-like plasmid (co-transferring *iro* and *iuc* loci, with or without *rmpA*/*rmpA2*) in 14/25 *K. pneumoniae* is predicted to confer a hypervirulent phenotype in these isolates [50, 51]. The *iuc* locus was also incorporated into plasmids highly similar to p205880-2FIIK (incFIB-incFII; accession number: MN824002), which also co-transfers *fecABCDRI* (Fe^3+^ uptake) and possibly enhanced the virulence of ST25, ST828 and ST193. Additionally, multiple ICE variants were responsible for the chromosomal integration of yersiniabactin biosynthesis cluster, as well as colibactin (ICE*Kp10*) or *iroABCDN-rmpA* (ICE*Kp1*) in some instances. The combination of these genetic elements underlines the high virulence of *K. pneumoniae* in our patient cohort. However, such virulent determinants were not detected in the remaining five infecting isolates (with two leading to fatal cases), suggesting the contribution of other unrecognized virulent factors.

**Table 2.** Summary of virulence profile in 25 infecting *K. pneumoniae* isolates from intensive care patients +/- indicates that the genetic element could be present (+) or absent (-) due to truncation/mutation (*rmpA* and *rmpA2*) or the acquisition of the respective ICE element (clb, iro, ybt). The siderophore includes enterobactin (ent), colibactin (clb), salmochelin (iro), aerobactin (iuc) and yersiniabactin (ybt). The number in parentheses indicates the number of isolates carrying the genetic element

ST	Infected patient	Hypermucoidy		Siderophores					Virulence plasmid	ICE	Mortality∗
		rmpA	rmpA2	ent	clb	iro	iuc	ybt			
23	K1, K22, K25	**+**	−	**+**	**+**	**+**	**+**	**+**	pLVPK-like	ICE*Kp10*	1/3
65	K3, K28	**+**	−	**+**	**+/-**	**+**	**+**	**+/-**	pLVPK-like	ICE*Kp10* (1)	0/2
420	K27	**+**	**+**	**+**	−	**+**	**+**	**+**	pLVPK-like	ICE*Kp3*	1/1
816-1LV†	K5	−	−	**+**	−	**+**	**+**	**+**	pLVPK-like	ICE*Kp4*	0/1
86	K6, K26	-**/+**	**+/-**	**+**	−	**+**	**+**	**+**	pLVPK	ICE*Kp3* (1), ICE*Kp12* (1)	1/2
	K8	**+**	−	**+**	−	**+**	**+**	−	pLVPK	−	0/1
25	K19	−	−	**+**	−	**+**	**+**	**+**	p205880-2FIIK	ICE*Kp1*	1/1
828	K14	**+**	−	**+**	−	**+**	**+**	**+**	Recombinant of p205880-2FIIK and pDA12090-1	ICE*Kp1*	0/1
592	K7, K12, K15	**+/-**	**+/-**	**+**	−	**+**	**+**	−	pLVPK-like	−	0/3
375	K4	−	−	**+**	−	**+**	**+**	−	pLVPK-like	−	1/1
17	K9, K11	−	−	**+**	−	**+/-**	−	**+**	−	ICE*Kp1* (1), ICE-unc (1)	1/2
15	K23	−	−	**+**	−	−	−	**+**	−	ICE*Kp4*	0/1
193	K13	−	−	**+**	−	−	**+**	−	p205880-2FIIK	−	0/1
Other‡	K17, K18, K20, K21, K24	−	−	**+**	−	−	−	−	−	−	2/5

*Interpreted as the number of reported death/total cases.

†*Klebsiella quasipneumoniae* isolate.

‡Other STs include ST35 (two cases), ST1215, ST1245 and a novel ST.

ICE: integrative conjugative element; ICE-unc: uncharacterized ICE.

The majority of infections (22/25) were susceptible to ceftriaxone, cefepime, amikacin, imipenem and piperacillin/tazobactam. Among these 25 isolates, non-susceptibility to other antimicrobials was infrequent, such as ciprofloxacin (*n*=1), co-trimoxazole (*n*=3), colistin (*n*=3, reduced susceptibility) and ticarcillin/clavulanate (*n*=5). In contrast, two infections were classified as XDR, and only susceptible to colistin (*n*=2), and amikacin (*n*=1) (Fig. 1b). These include an ST15 carrying the carbapenemase-producing plasmid pKP27-NDM4 *[aac(3)-Iid*, *aac(6′)-lb*, *aadA1*, *qnrS1*, *bla*_LAP-2_, *bla*_OXA-9_, *bla*_CTX-M-14_, and *bla*_NDM-4_; accession number: NZ_CP041642], which was previously isolated in HCMC, Vietnam [52]; and an ST17 carrying pCFI-3 *[aac(6′)-lb-cr*, *arr-3*, *bla*_OXA-1_, *sul1*, *catB3*, *qnrB4*, and *bla*_DHA-1_; accession number: NC_019984]. The presence of *bla*_DHA-1_ in several ST17 and ST86 strains underlay their resistance to ceftriaxone and ticarcillin/clavulanate. On the other hand, carriage of *bla*_OXA-1_ coincides with resistance to cefepime and piperacillin/tazobactam, in line with previous reports [53, 54]. ST23 was pan-susceptible to all tested antimicrobials, except for one being resistant to ticarcillin/clavulanate and another with reduced susceptibility to colistin (Fig. 3c). We reported eight fatal *K. pneumoniae* infections (three BSIs, three pneumonia, two SBPs) in this study, but the patient’s outcome is not associated with MDR status or accumulation of siderophores in the infecting isolate (Fisher's exact test, *P*>0.05).

## Discussion

Our research is among the few studying the link between colonization and infection of *S. aureus* and *K. pneumoniae* in resource-limited ICU settings. Previous studies, mostly from high-income settings, found that the colonizing and infecting bacterial isolates were frequently of similar genotypes. A landmark study conducted in Germany revealed that >80 % of bacteraemia-causing *S. aureus* were clonally identical, by PFGE typing, to those colonizing the nares [55]. Concordance in MLST and antibiogram was also noted between colonizing and infecting *S. aureus* recovered in children in Korea [17]. Utilizing MLST comparisons, our study found that the ST of the infecting isolate frequently matched that of colonizing isolates in the respective patients, and this observation was consistent for all reported HAIs but variable among CAIs. This suggested that the infections might be caused by the *S. aureus* and *K. pneumoniae* colonizing the host prior to disease onset, but high-resolution genomic approaches should be used to confirm this connection. For instance, core genome MLST (634 genes) was implemented to confirm the identity between rectal colonizing and infecting *K. pneumoniae* in the USA [19], while detailed genomic comparison confirmed that ~50 % of *K. pneumoniae* infections were caused by the patient’s own colonizing strain in an Australian ICU setting [20].

ST188 was the predominant *S. aureus* clone reported in our study. Our high-resolution genomic analysis pointed to the genetic clonality of infecting and colonizing ST188 in 6/7 patients, confirming that preceding colonization at the nasal and/or endotracheal sites could directly cause infections. Although it is not yet recognized as a pandemic clone, ST188 has increasingly been isolated from infections in the Asia–Pacific region. While most reported infections in China and Taiwan were methicillin-sensitive [56, 57], MRSA ST188 is becoming prevalent in Hong Kong and Malaysia [58, 59]. Phylogenetic analysis has proposed that ST188 first emerged in livestock in the 1960s, subsequently spreading to other animals and humans, forming the current clinically significant ST188 clone [56]. Experimental evidence indicates that ST188 shows enhanced epithelial cell adhesion and biofilm formation, which explains its high frequency in nasal colonization in patients [56]. In Vietnam, ST188 has been reported previously in the Northern, Central and Southern regions [60, 61]. The ST188 recovered in our study was both highly virulent and MDR, causing both CAIs and HAIs. Therefore, future surveillance efforts are warranted to evaluate the prevalence and risk of ST188 carriage in Vietnam.

In addition, we reported that *S. argenteus* colonization led to infections in three patients, supported by the high genetic similarity between infecting and colonizing isolates. A conventional microbiology approach could not distinguish *S. argenteus* from *S. aureus *, as the two species share numerous biochemical characteristics [62, 63], such as catalase- and coagulase-positivity. Thus, molecular techniques, such as WGS and MS [64], could be implemented to offer accurate classification, in order to estimate the clinical burden of *S. argenteus *. Although it was proposed to be less virulent due to the lack of staphyloxanthin [65], *S. argenteus* has been associated with community impetigo in Australia [66] and CAI in Thailand [63]. The two genotypes (ST2250 and ST1223) reported herein have been detected previously in Thailand [67] and Japan [48], with the former being more widespread. Similar to our findings, a recent surveillance reported that *S. argenteus* isolates in Thailand were susceptible to most antimicrobials and harboured fewer virulence genes [67]. Clinical investigation concluded that, as compared to *S. aureus *, *S. argenteus* was less likely to induce respiratory failure in patients, although this did not result in a difference in mortality [67].

We reported a great diversity of *K. pneumoniae* causing infections in our cohort. In-depth genomic analysis of ST17, ST23, ST25 and ST86 revealed that the infecting strain was genetically similar to colonizing strains in the same patient. ST23 (KL1 type) and ST86 (KL2 type) are known hypermucoviscous clones with a propensity to cause CA invasive diseases [68, 69], while ST17 (with diverse capsular types) is more elusive. ESBL-producing ST17 has been associated with a neonatal ICU outbreak in Norway in 2008–2009 [70], showing that it is of clinical interest. In contrast to *S. aureus *, the isolated *K. pneumoniae* (both colonizing and infecting) remain susceptible to most antimicrobials, although possessing a high degree of virulence potential via the acquisition of a hypervirulence plasmid and yersiniabactin-encoding ICEs. This observation is congruent with recent systemic analysis, demonstrating that highly virulent *K. pneumoniae* are usually more susceptible to antimicrobials [71], as seen with ST23 recovered from this study. Nevertheless, the epidemic of carbapenem-resistant hypervirulent ST11 in China has raised the concern of novel, deadly and highly resistant *K. pneumoniae* clones in Asian countries [72].

Although measures to reduce cross-transmission between patients are important in nosocomial settings, our results emphasize the importance of minimizing the risk of infections from the patients’ own microbiome [73, 74]. So far, *S. aureus* decolonization of the nares and other body sites has been implemented more extensively. Use of nasal mupirocin has provided the most favourable effects, active against a wide range of staphylococci, including MRSA [75, 76]. On the other hand, digestive decontamination has been studied extensively in ICU patients to prevent or eradicate the oropharyngeal and intestinal carriage of pathogenic *K. pneumoniae *, but this approach risks damaging the gut microbiota homeostasis [77]. The use of these decolonization agents has been limited to some ICUs in Europe and North America, where the extent of AMR in the microbiomes is lower. However, selective digestive decontamination is among the few interventions in ICUs which has shown reductions in infection rates in critically ill patients and improved outcome [77]. It represents a possible approach in LMIC settings, such as Vietnam, where AMR is on the rise and there are not many effective solutions. However, the selection of active agents is likely to be difficult, and the major risk is that it may promote colonization with the most resistant bacteria.

Our research combined the strength of detailed clinical investigation and high-resolution genomic analysis on a wealth of colonizing *S. aureus* and *K. pneumoniae* obtained prior to disease onset. This provided comprehensive tracking of disease progression, thus allowing us to confidently assess the colonization–infection causality. There are some inherent limitations in our study. Some colonizing and infecting isolates could not be retrieved for WGS, and we did not exhaustively perform genomic comparison on all STs. Besides, the sampling for CAI was not intensive due to the narrow sampling timeframe. Our study did not include other patients admitted to the ICU during the study period, so it was not possible to fully document the event of cross-transmission from these patients. Therefore, it is likely that we have underestimated the contribution of colonization to infection in the two pathogens. Although our study site (a referral hospital for infectious diseases in Southern Vietnam) could capture patients from a wide geo-social background, the findings may not represent the circumstance in other hospitals in the region.

In summary, our study highlights that carriage of *S. aureus* or *K. pneumoniae* possessing multiple virulence determinants directly leads to development of invasive diseases in patients in an Asian ICU setting. This insight calls for more effective and innovative approaches to manage the risk of infection, preferably coupled with rigorous microbiome screening targeting the bacterial virulence determinants. Future studies should identify which virulence factors are of clinical significance and feasible interventions that can be implemented in low-resource settings.

## Supplementary Data

Supplementary material 1

Supplementary material 2
