# Supplementary material for: Colonization with Staphylococcus aureus and Klebsiella pneumoniae causes infections in a Vietnamese intensive care unit
Source: Microb Genom. 2021 Jan 27;7(2):000514. doi: 10.1099/mgen.0.000514 (PMC8208697; doi:10.1099/mgen.0.000514)
Supplement: Supplementary material 2 [file mgen-7-514-s002.pdf]

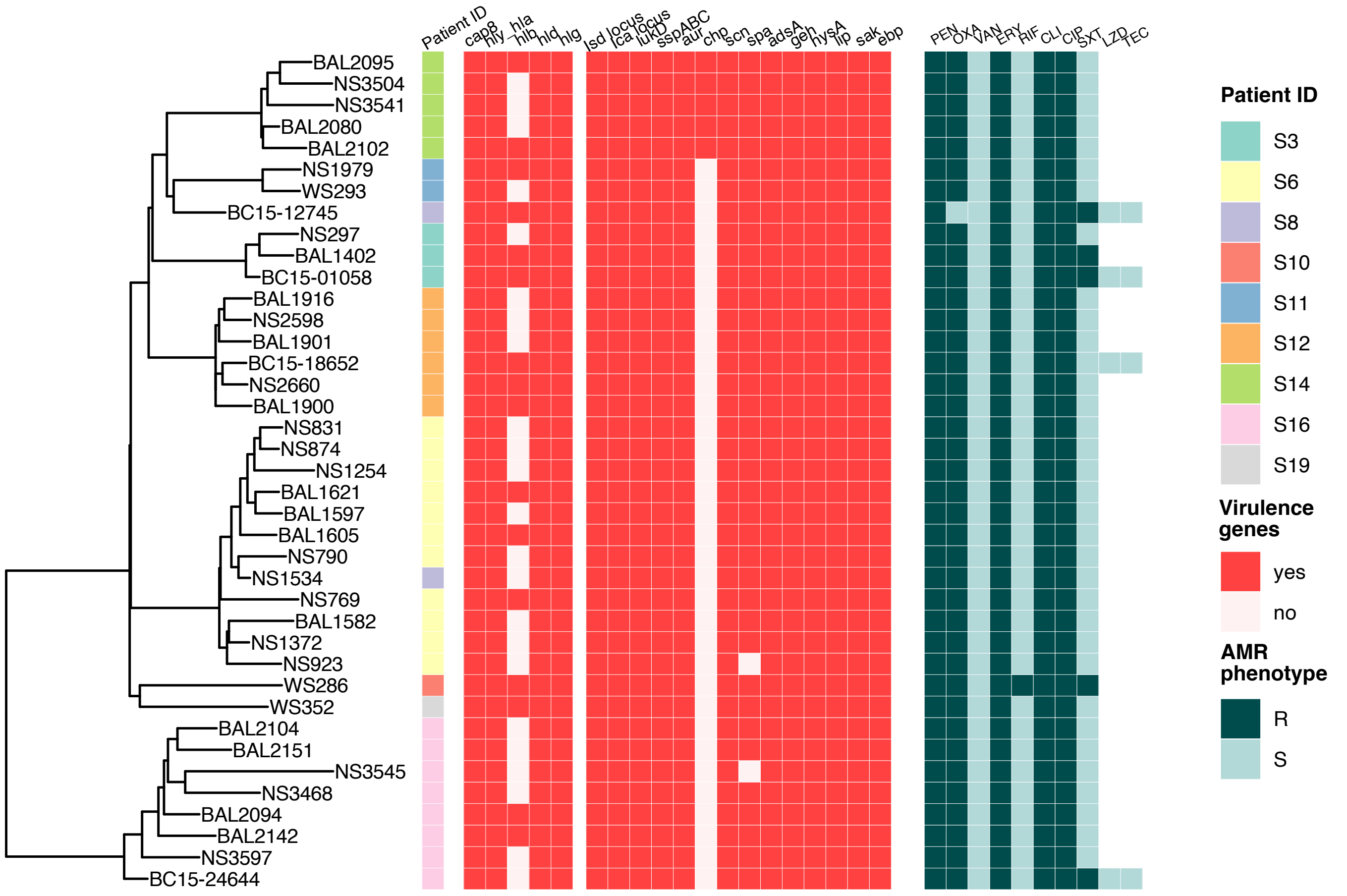

Figure S1 The virulence gene and antimicrobial susceptibility profiles of *Staphylococcus aureus* ST188. The phylogeny is identical to that described in Figure 2B. The columns show the associated information for each taxon, including Patient ID; the presence of virulence genes, including type 8 capsule (*cap8*), hemolysins (*hly_hla*, *hlb*, *hld*, *hlg*), leucocidin D (*lukD*), haem uptake (*isd* locus), adhesion factors (*ica* locus, *ebp*), immune escape (*scn*, *spa*, *adsA*, *sak*), chemotaxis-inhibiting protein (*chs*), serine protease (*sspABC*, *aur*), lipases (*geh*, *lip*), hyaluronidase (*hysA*); susceptibility to antimicrobials, including PEN (penicillin), OXA (oxacillin), VAN (vancomycin), ERY (erythromycin), RIF (rifampicin), CLI (clindamycin), CIP (ciprofloxacin), SXT (co-trimoxazole), LZD (linezolid), and TEC (teicoplanin). R: Resistant, S: Susceptible.

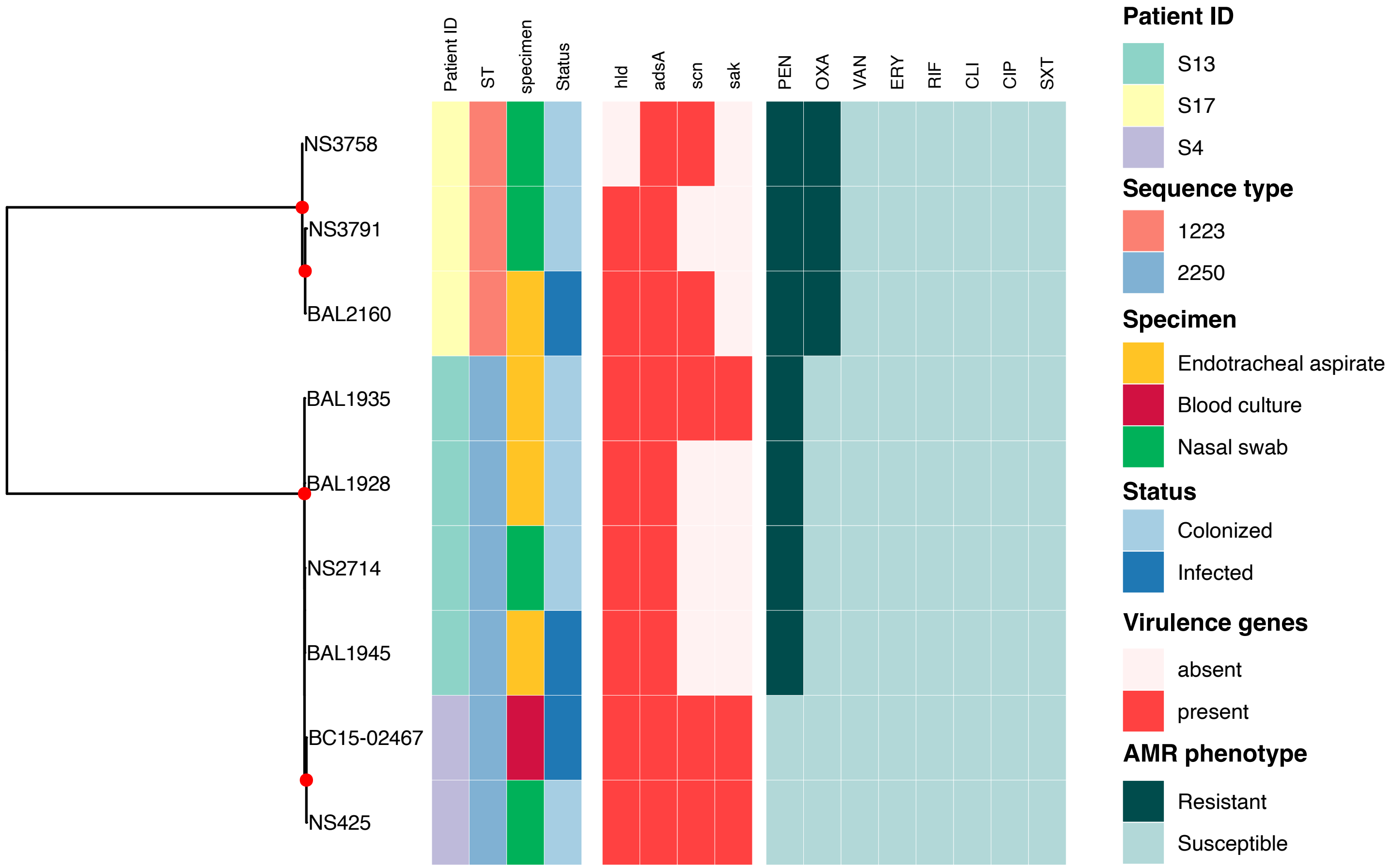

Figure S2 Genomic investigation of Staphylococcus argenteus causing infections in this study. The maximum likelihood phylogeny was constructed using recombination-free SNP alignment (output from Gubbins) after mapping to the reference ASM23692 (See Methods). The tree is midpoint rooted, and the red filled circles indicate bootstrap values greater than 80 at the internal nodes. The columns show the associated information for each taxon, including Patient ID, sequence type, specimen and source of isolation (colonizing or infecting); the presence of virulence genes, including hemolysins (*hld*), immune escape (*scn*, *adsA*, *sak*); susceptibility to antimicrobials, including PEN (penicillin), OXA (oxacillin), VAN (vancomycin), ERY (erythromycin), RIF (rifampicin), CLI (clindamycin), CIP (ciprofloxacin), SXT (co-trimoxazole).
